# Adenoviral delivery of pan-caspase inhibitor p35 enhances bystander killing by P450 gene-directed enzyme prodrug therapy using cyclophosphamide^+^

**DOI:** 10.1186/1471-2407-10-487

**Published:** 2010-09-13

**Authors:** Joshua C Doloff, Ting Su, David J Waxman

**Affiliations:** 1 Division of Cell and Molecular Biology Department of Biology, Boston University, Boston, MA 02215, USA

## Abstract

**Background:**

Cytochrome P450-based suicide gene therapy for cancer using prodrugs such as cyclophosphamide (CPA) increases anti-tumor activity, both directly and *via *a bystander killing mechanism. Bystander cell killing is essential for the clinical success of this treatment strategy, given the difficulty of achieving 100% efficient gene delivery *in vivo *using current technologies. Previous studies have shown that the pan-caspase inhibitor p35 significantly increases CPA-induced bystander killing by tumor cells that stably express P450 enzyme CYP2B6 (Schwartz *et al*, (2002) Cancer Res. 62: 6928-37).

**Methods:**

To further develop this approach, we constructed and characterized a replication-defective adenovirus, Adeno-2B6/p35, which expresses p35 in combination with CYP2B6 and its electron transfer partner, P450 reductase.

**Results:**

The expression of p35 in Adeno-2B6/p35-infected tumor cells inhibited caspase activation, delaying the death of the CYP2B6 "factory" cells that produce active CPA metabolites, and increased bystander tumor cell killing compared to that achieved in the absence of p35. Tumor cells infected with Adeno-2B6/p35 were readily killed by cisplatin and doxorubicin, indicating that p35 expression is not associated with acquisition of general drug resistance. Finally, p35 did not inhibit viral release when the replication-competent adenovirus ONYX-017 was used as a helper virus to facilitate co-replication and spread of Adeno-2B6/p35 and further increase CPA-induced bystander cell killing.

**Conclusions:**

The introduction of p35 into gene therapeutic regimens constitutes an effective approach to increase bystander killing by cytochrome P450 gene therapy. This strategy may also be used to enhance other bystander cytotoxic therapies, including those involving the production of tumor cell toxic protein products.

## Background

Gene-directed enzyme prodrug therapy (GDEPT) for cancer is designed to increase the chemotherapeutic sensitivity of tumor cells by introduction of a gene coding for a prodrug activation enzyme. Using this approach, a prodrug-activating gene is delivered to tumor cells in a selective manner, e.g., using a tumor-selective viral vector carrying the therapeutic gene [1-4]. Tumor cells that express the prodrug-activating gene acquire the capacity to convert a prodrug to its active cytotoxic metabolite, leading to cell death upon contact with the prodrug [5]. GDEPT thus provides the potential to improve cancer therapy by sensitizing tumor tissue to a chemotherapeutic prodrug. Ultimately, this strategy may allow for a reduction in drug dosages needed to achieve therapeutic activity, thereby decreasing systemic side effects towards critical host tissues, such as bone marrow, kidney and heart [6,7].

Commonly studied GDEPT systems include herpes simplex virus thymidine kinase (HSV-tk) in combination with the prodrug ganciclovir, *E. coli *cytosine deaminase with 5-fluorocytosine, and cytochrome P450 with cyclophosphamide (CPA) or ifosfamide [8]. P450-based GDEPT has several advantages: 1) P450 is a multiple enzyme-multiple drug system, unlike other GDEPT systems, which are essentially one enzyme-one drug systems [8-11]; 2) P450 GDEPT can be implemented using established anti-cancer agents, such as CPA and ifosfamide, as well as investigational agents, such as the CYP3A4 prodrug methoxymorpholinyl doxorubicin [12], the CYP1A2 prodrug dacarbazine [13], 4-ipomeanol, ftorafur, and tamoxifen, among others [7,8]. Moreover, P450 GDEPT may be useful in treating a broad spectrum of cancers, including breast cancer, melanoma, pancreatic cancer, and metastatic liver cancer [14-17]; 3) mammalian P450 subfamily 2B enzymes, in particular rat CYP2B1 [18], human CYP2B6 [10,11], and dog CYP2B11 [19,20], are effective catalysts of CPA activation. The use of a P450 gene of mammalian origin reduces the likelihood of inducing adverse immune responses; 4) the active metabolites of P450 prodrugs, such as CPA, can readily diffuse from cell to cell via non-facilitated mechanisms, conferring a strong bystander effect even in the absence of direct cell-cell contact [8,21,22], in contrast to certain other prodrugs used for GDEPT, such as ganciclovir [23]. Bystander killing is an essential feature of any GDEPT system, insofar as it helps circumvent the requirement (unachievable using current gene delivery technologies) to transduce 100% of the target tumor cell population with the therapeutic gene.

Conditionally replicating adenoviruses offer the advantage of selective replication in cancer cells and are commonly used as gene delivery vectors [4,5]. A prototypical example is the adenovirus ONYX-015 and its closely related derivative ONYX-017 (E3 region wild-type), both expected to replicate in p53-defective cells [24,25]. These replicating adenoviruses can be combined with replication-defective Adeno-P450 viruses to facilitate therapeutic delivery of P450, or other therapeutic genes, in tumor cells *in vivo *[20,24]. This combination of conditionally replicating and non-replicating adenoviruses could be ideal for GDEPT, due to the synergistic effect of combining replicating virus-induced tumor cytolysis with intratumoral activation of chemotherapeutic prodrugs conferred by the replication-defective virus.

One gene therapeutic approach to increasing tumor cell kill involves the introduction of pro-apoptotic factors to augment drug-induced tumor cell apoptosis. This approach has been exemplified using the pro-apoptotic factors Bax, p53, Trail and various caspases, and has been investigated in both preclinical and clinical studies, either alone or in combination with traditional chemotherapy [26]. However, a serious limitation of this strategy is that it does not elicit bystander cytotoxicity, and consequently, the pro-apoptotic gene must be introduced into the tumor cell population *in vivo *with an efficiency approaching 100% to achieve an effective and sustained anti-tumor response. Furthermore, pro-apoptotic factor-based therapies are not suitable for combination with GDEPT, as they undermine the bystander killing effect that is essential for tumor cell eradication [26]. An alternative, albeit counter-intuitive approach combines GDEPT with the introduction of anti-apoptotic factors, and is designed to prolong the longevity of those tumor cells that produce the prodrug-activating enzyme, allowing them to generate an increased amount of cytotoxic prodrug metabolites, but in a way that does not ultimately block the death of those tumor cells [26]. This strategy was initially investigated using caspase inhibitors to delay the death of tumor cells carrying a prodrug-activating P450 gene. In a comparison of four caspase inhibitors, the baculovirus protein p35, a 35-kDa single chain broad-spectrum caspase inhibitor [27,28], was found to be the most effective in delaying the death of CPA-treated 9L gliosarcoma cells stably expressing P450 [29]. Importantly, the introduction of p35 delayed, but did not block, the ultimate death of the transduced tumor cells and thereby increased the bystander killing effect of CPA. The present study further develops this strategy with the introduction of a replication-defective adenovirus that co-expresses the prodrug-activating P450 enzyme CYP2B6 and the pan-caspase inhibitor p35, and is shown to augment bystander killing and the overall anti-cancer response.

## Methods

### Cell lines and reagents

CPA was purchased from Sigma-Aldrich (St. Louis, MO). Fetal bovine serum (FBS) and RPMI 1640 medium were purchased from Invitrogen (Frederick, MD). Human tumor cell lines U251 (brain) and A549 (lung) were obtained from Dr. D. Scudiero (National Cancer Institute, Bethesda, MD). The rat 9L gliosarcoma tumor cell line was obtained from the UCSF Neurosurgery Tissue Bank (San Francisco, CA). Tumor cells were grown at 37°C in a humidified, 5% CO_2 _atmosphere in RPMI 1640 culture medium containing 5% FBS, penicillin (100 units/ml), and streptomycin (100 μg/ml). The conditionally replicating adenovirus ONYX-017 [24], which contains a wild-type viral E3 region and an E1B-55 kDa gene deletion, was obtained from ONYX Pharmaceuticals (Richmond, CA).

### Construction of Adeno-2B6/p35

Virus was constructed in the following three steps. (1) Subcloning of p35 into pORF, to create a p35 expression cassette: p35 cDNA was excised with EcoR I from pBluescript-p35, obtained from Dr. Thomas D. Gilmore (Boston University, Boston, MA), and cloned into pORF-MCS (InvivoGen, San Diego, CA) linearized with EcoR I. The resulting plasmid, pORF-p35, was digested with Bgl I to select for clones with the correct orientation. (2) Subcloning of p35 expression cassette into pShuttle-2B6-IHOR: To obtain a blunt-ended p35 expression cassette driven by a hEF1-HTLV promoter, pORF-p35 was digested with BfuA I then blunt-ended using Klenow enzyme. The linearized plasmid was then digested with Swa I. To clone the p35 expression cassette into pShuttle-2B6-IHOR, which contains CYP2B6 cDNA in linked to P450 reductase cDNA *via *an internal ribosome entry sequence [24], pShuttle-2B6-IHOR was first digested with Mlu I, dephosphorylated, and blunt-ended with Klenow enzyme. The p35 expression cassette and pShuttle-2B6-IHOR vector, both gel purified, were ligated to create pShuttle-2B6-IHOR-p35. (3) Subcloning of 2B6-IHOR-p35 into Adeno-X: To obtain the final recombinant adenoviral DNA, a 2B6-IHOR-p35 fragment was excised from pShuttle-2B6-IHOR-p35 by PI-Sce I/I-Ceu I double digestion followed by ligation with Adeno-X viral DNA linearized using the same enzymes. The reaction mixture was then digested with Swa I to eliminate non-recombinants. Clones containing the 2B6-IHOR-p35 fragment were identified by PCR using CYP2B6 primers. Adenovirus was then generated following the manufacturer's instructions (Clontech, Mountain View, CA, USA). The recombinant Adeno-X-2B6-IHOR-p35 was digested with Pac I, and the linearized viral plasmid was transfected into low passage HK293 cells. A cytopathic effect was apparent after 10-14 days, at which point Adeno-2B6/p35 virus was isolated, amplified in HEK293 cells, purified, and quantified using the Adeno-X Rapid Titer kit (Clontech Labs), as described in the manufacturer's protocol [24].

### qPCR analysis

qPCR was performed as described [24] using gene-specific primers for quantification of CYP2B6 (sense: 5'-ACTGCTCTCCATGACCCACACT-3' and antisense: 5'-CGATGCCTTCACCAAGACAAA-3') and p35 cDNA (sense: 5'-TCGACGAACGCAACGACTAC-3' and antisense: 5'-CTTGGTTGCTGCCGTTCTC-3').

### Western blotting

Cell extracts (20 μg) prepared in lysis buffer (20% glycerol, 1% Triton X-100, 20 mM HEPES (pH 7.9), 1 mM EDTA, 1 mM EGTA, 20 mM NaF, 1 mM Na_4_P_2_O_7_, 1 mM DTT, 1 mM Na_3_VO_4_, 1 mg/ml leupeptin, and 1 mg/ml pepstatin) were analyzed by Western blotting. CYP2B6 protein was detected using a monoclonal rabbit anti-P450 2B6 antibody (1:3000 dilution; Gentest Corp., Woburn, MA), and p35 protein was detected using polyclonal rabbit anti-p35 antibody (1:3000 dilution; Biocarta, CA). Goat anti-rabbit HRP-conjugated secondary antibody (1:3000 dilution) was used for both primary antibodies (Amersham). Blots were visualized with enhanced chemiluminescence (ECL) Western blotting detection reagent (Amersham Pharmacia Biotech) exposed to Kodak X-OMAT blue film XB-1, and scanned to verify equal expression of CYP2B6 in tumor cells infected with Adeno-2B6 and Adeno-2B6/p35.

### Quantification of cellular 4-OH-CPA production

U251 cells were plated at 100,000 cells/well with 1 ml medium/well in a 12-well plate. 24 hr later, cells were infected with Adeno-2B6 or Adeno-2B6/p35 at multiplicities of infection (MOIs) of 0, 3.75, 7, and 15. Cellular CPA 4-hydroxylase activity was determined 48 hr and 72 hr post-infection by 4-OH-CPA released into fresh culture medium containing 1 mM CPA over a 4 hr period. 5 mM semicarbazide was included in the medium to trap and stabilize the initial 4-OH-CPA metabolite. An aliquot of medium (0.5 ml) was removed from each well and snap frozen in liquid nitrogen and stored at -80°C until processing and HPLC analysis as previously described [20]. Cells remaining on the plate were washed with 1X PBS and stained with crystal violet (A595) to normalize 4-OH levels to cell content for calculation of cellular CPA 4-hydroxylase-specific activity (nmol 4-OH-CPA/ml media/A595). HPLC calibration curves were generated using standard curves of 4-OH-CPA metabolite prepared using 4-OOH-CPA dissolved in cell culture medium [20].

### Caspase activity assay

Caspase-3/7 activities were measured using the Apo-ONE homogeneous caspase-3/7 assay (Promega, Madison, WI). Cells were plated in a 96-well plate at a density of 1,000 cells/0.1 ml/well. 24 hr after cell seeding, cells were infected with Adeno-2B6 or Adeno-2B6/p35 at MOIs specified in each figure. 48 hr after viral infection, medium was aspirated and 1 mM CPA was added in a vol of 60 μl/well. 48 hr after beginning drug treatment, caspase-3/7 activity was assayed by adding an equal volume (60 μl) of homogeneous caspase-3/7 reagent to each well, and the plate was incubated for 2 hr at room temperature with constant shaking at 300 rpm. Fluorescence was measured with an ABI Prism 7900HT sequence detection system (Applied Biosystems, Foster City, CA) using an allelic discrimination method with rhodamine 110 as a reporter dye. The value of bin 7, which is the peak of the raw fluorescence curve, was used to indicate the activities of caspase-3/7.

### Immunohistochemical analysis and TUNEL staining

For immunohistochemistry, cells were fixed for 20 min with 4% formaldehyde and permeabilized with 1% Triton-X100/1% sodium citrate for 5 min at 4°C. Blocking was performed using 2% normal goat serum for 1 hr at room temperature. Cells were stained with 1:2000 diluted mouse anti-human 2B6 monoclonal antibody (GenTEST, BD Biosciences, Cat. #458326) for 1 hr at room temperature. Secondary antibody incubation used HRP-conjugated anti-mouse IgG (1:500 dilution) at room temperature for 1 hr. DAB or VIP was used as the substrate for HRP. TUNEL staining was used to assay the effect of p35 on CPA-induced apoptosis, as follows. 6,000 U251 cells were seeded in two 4-well chamber slides. The next day, cells were infected with Adeno-2B6 or Adeno-2B6/p35 at MOIs of 0 and 25. 48 hr post-infection, 1 mM CPA was added to each well for 48 hr. Slides were then fixed with 4% paraformaldehyde and processed with the DeadEnd colorimetric TUNEL system (cat. no. G7360; Promega). For double staining, immunohistochemistry for CYP2B6, as above, was carried out following TUNEL staining.

### Growth inhibition assay

To assay the effect of p35 on CPA-induced cell death, 15,000 U251 cells were plated in 24-well plates 24 hr prior to viral infection. The next day, cells were infected with either Adeno-2B6 or Adeno-2B6/p35 at MOIs 0, 2.5, 5, and 10 in a minimal volume of 200 μl for 3 hr, following which 1 ml of fresh medium was added/well. 48 hr after viral infection, either no drug or 1 mM CPA was added to the cells for 4 days. Cells were stained with crystal violet and quantified by measuring the 70% ethanol-eluted crystal violet (A595). To assay cisplatin and doxorubicin cell sensitivity, U251 cells were seeded in a similar fashion but were infected with either virus at MOIs 15 and 30 and then treated for 4 days at 0, 5, 10, 20, and 40 μM (cisplatin) or at 0, 20, 40, 80, and 160 nM (doxorubicin).

### Bystander killing

Since 9L cells are virtually uninfectable by adenovirus at MOIs ≤100 (data not shown), 9L cells were used as uninfectable bystander cells. To assay bystander killing, U251 and 9L/LacZ cells were mixed at a ratio of 60:40 then plated at 150,000 cells/well of a 12-well plate. 24 hr later the cells were infected with either Adeno-2B6 alone, Adeno-2B6/p35 alone, Adeno-2B6 + ONYX-017, or Adeno-2B6/p35 + ONYX-017 at MOIs calculated based on the number of seeded U251 cells, as indicated in the figures. 48 hr after viral infection, mixed cells were treated with 1 mM CPA for either 8 or 24 hr. A second treatment with 1 mM CPA for 8 or 24 hr was applied to the cells 48 hr after the beginning of the first CPA treatment. 16 hr after the end of the second CPA treatment, cells were trypsinized, counted, and reseeded at various densities in 6-well plates to allow for the growth of individual colonies, as follows: drug-free control cells were replated in duplicate at 100, 200, and 400 cells/well; and CPA-treated cells were replated at 1,000, 2,000, and 4,000 cells/well. Cells were grown for 7-9 days, and then stained with X-Gal. LacZ-positive colonies containing ≥50 cells were counted. The efficiency of colony formation was then calculated [29].

### ONYX-017 helper virus studies

To determine whether expression of p35 from Adeno-2B6/p35 interferes with the replicating virus helper effect or viral spread by ONYX-017, U251 and A549 cells were plated at 8,000 cells/well of a 24-well plate, and 24 hr later the cells were infected for 4 hr with either Adeno-2B6, Adeno-2B6/p35, Adeno-2B6 + ONYX-017, or Adeno-2B6/p35 + ONYX-017 (MOI 5 for Adeno-2B6 and Adeno-2B6/p35, and MOIs of 0, 1, or 3 for ONYX-017). Virus was then removed and fresh medium was added to each well. Cell supernatants were isolated on days 1, 2, 3, 4, 5, and 6 post viral infection, and qPCR was performed on aliquots to assay for viral presence using primer sets for CYP2B6 and p35 cDNA, as described above. Viral particle numbers were determined by normalization to a calibration curve generated by qPCR analysis of known viral dilutions. To assay the ultimate extent of p35 bystander activity augmentation via the viral helper effect, 7,000 U251 + 9L/lacZ cells (ratio of 40:60) were seeded in a 12-well plate. The following day, cells were infected with ONYX-017 (MOIs of 0, 1, and 3) in combination with either Adeno-2B6 or Adeno-2B6/p35 (MOIs of either 7.5 or 15, using MOI values calculated based on initial U251 cell numbers). After a 48 hr viral incubation, either no drug or 1 mM CPA was added to the cells for 48 hr. 48 hr later the cells in each well were trypsinized, counted and replated at densities of 1,000 and 3,000 cells in two wells of a 6-well plate. Colony formation then proceeded for 5-7 days, after which the cells were stained with X-gal and counted to determine the impact of p35 expression on bystander activity, as judged by a decrease in tumor cell colony formation.

### Data analysis

Data is presented as mean ± SD based on either technical duplicates or triplicates, as indicated. In addition, to ensure that all findings were reproducible and representative, experimental results were confirmed in at least 2 or 3 independent sets of experiments.

## Results

### Characterization of Adeno-2B6/p35

**Figure 1:**
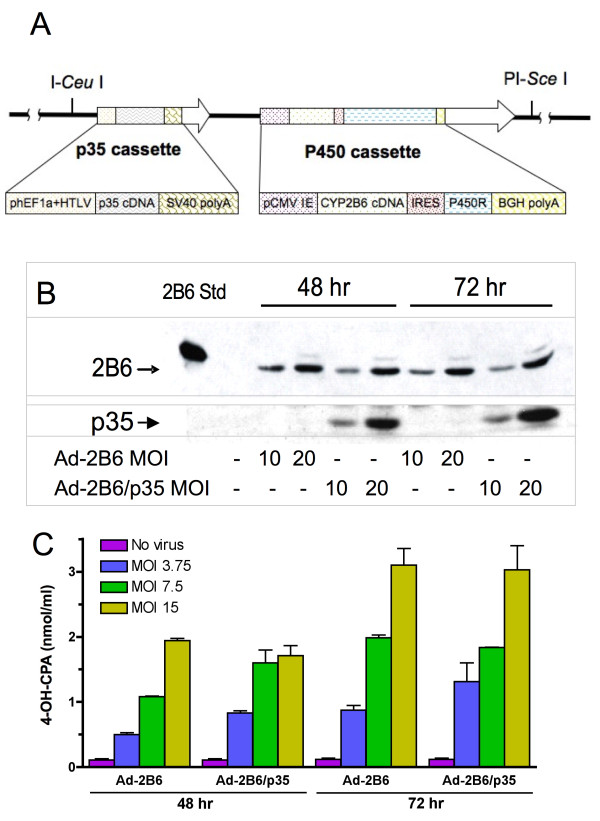
**Design and characterization of Adeno-2B6/p35**. A) Schematic showing separate p35 and P450 expression cassettes introduced into Adeno-2B6/p35. The P450 cassette encodes CYP2B6 linked to P450 reductase cDNA *via *an internal ribosome entry sequence ('IRES') and was originally derived from Adeno-2B6 [24]. B) Western blots showing CYP2B6 (*top*) and p35 (*bottom*) protein in 293 cells infected for 48 or 72 hr with either Adeno-2B6 and Adeno-2B6/p35/p35 at MOI 0, 10 or 20, as indicated. Importantly, the level of CYP2B6 protein was similar in cells infected with Adeno-2B6 and Adeno-2B6/p35. C) Functional activity of the CYP2B6-expressing adenoviruses, shown by HPLC analysis of 4-OH-CPA production by U251 cells infected with Adeno-2B6 or Adeno-2B6/p35 (MOIs 0, 3.75, 7, and 15; 48 and 72 hr post-infection) after incubation with 1 mM CPA for 4 hr (mean ± SD, n = 2).

Adeno-2B6/p35 was prepared by introducing the pan-caspase inhibitor p35 into Adeno-2B6, which expresses P450 enzyme CYP2B6 and its redox partner P450 reductase [24] (Figure 1A). Lysates of 293 cells infected with Adeno-2B6 or Adeno-2B6/p35 contained similar levels of CYP2B6 protein 48 and 72 hr post infection (Figure 1B, *top*). p35 protein was only detected in cells infected with Adeno-2B6/p35 (Figure 1B, *bottom*). qPCR analysis confirmed similar expression levels of CYP2B6 in cells infected with each virus, whereas p35 transcripts were only detectable in the Adeno-2B6/p35-infected cells (*data not shown*). To verify that the expressed CYP2B6 protein is active, supernatants from 48 or 72 hr virus-infected U251 cells were pulsed with CPA and assayed for virus-dependent formation of 4-OH-CPA, the active metabolite of CPA. Conversion of CPA to 4-OH-CPA proceeded to a similar extent in cells infected with either virus (Figure 1C), demonstrating that both adenoviruses are functionally equivalent in producing similar levels of CYP2B6 metabolic activity.

### p35 inhibition of CPA-induced apoptotic cell death

**Figure 2:**
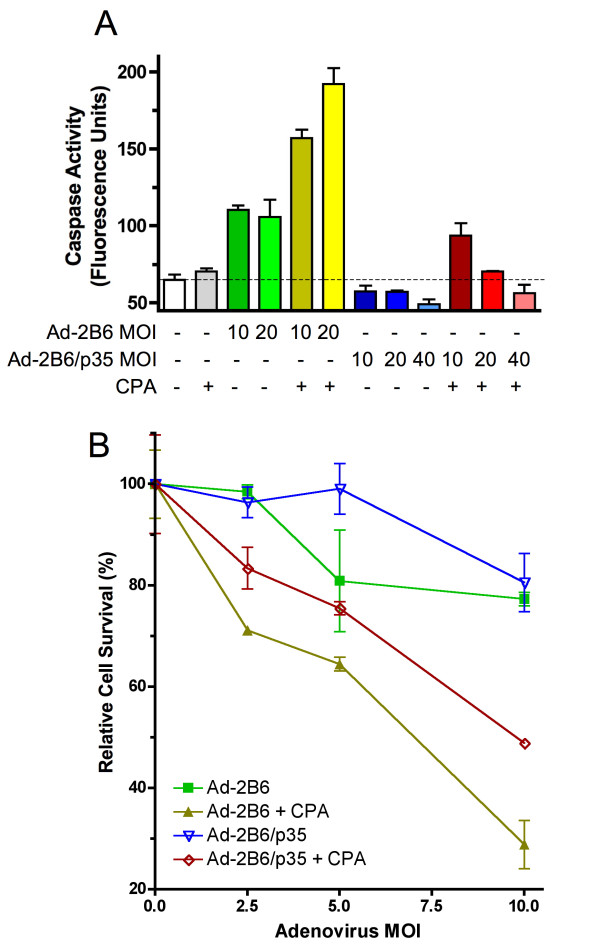
**CPA-induced cell death is inhibited by adenovirus delivered p35**. A) Caspase activity in U251 cells infected by Adeno-2B6 or Adeno-2B6/p35 for 48 hr (MOIs 0-40, as indicated) and treated with 1 mM CPA for 48 hr, as specified. Adeno-2B6 increased and Adeno-2B6/p35 decreased basal, and CPA-induced caspase activity in a virus dose-dependent manner. Caspase activity is expressed in arbitrary fluorescent units, with a dashed horizontal line indicating the basal caspase activity of uninfected cells in the absence of CPA treatment. B) Growth inhibition assay showing that CPA-induced U251 cell death is decreased in cells infected with Adeno-2B6/p35, as compared to cells infected with Adeno-2B6 (mean ± SD, n = 3, based on crystal violet staining quantified by A595).

Next, we investigated the functionality of p35 expressed in the adenovirus-infected tumor cells, as judged by its inhibition of both basal and CPA-induced caspase activity. Figure 2A shows that p35 markedly suppressed tumor cell caspase activity induced by a 48 hr adenoviral infection, as seen from a comparison of caspase activity in extracts of U251 human brain tumor cells infected with Adeno-2B6 vs. Adeno-2B6/p35. Moreover, p35 blocked in a dose-dependent manner the increase in caspase activity when the Adeno-2B6/p35-infected cells were further treated with CPA (Figure 2A). Furthermore, overall tumor cell survival was increased following CPA treatment in cells infected with Adeno-2B6/p35 as compared to Adeno-2B6 (Figure 2B).

### p35 does not induce global drug resistance

**Figure 3:**
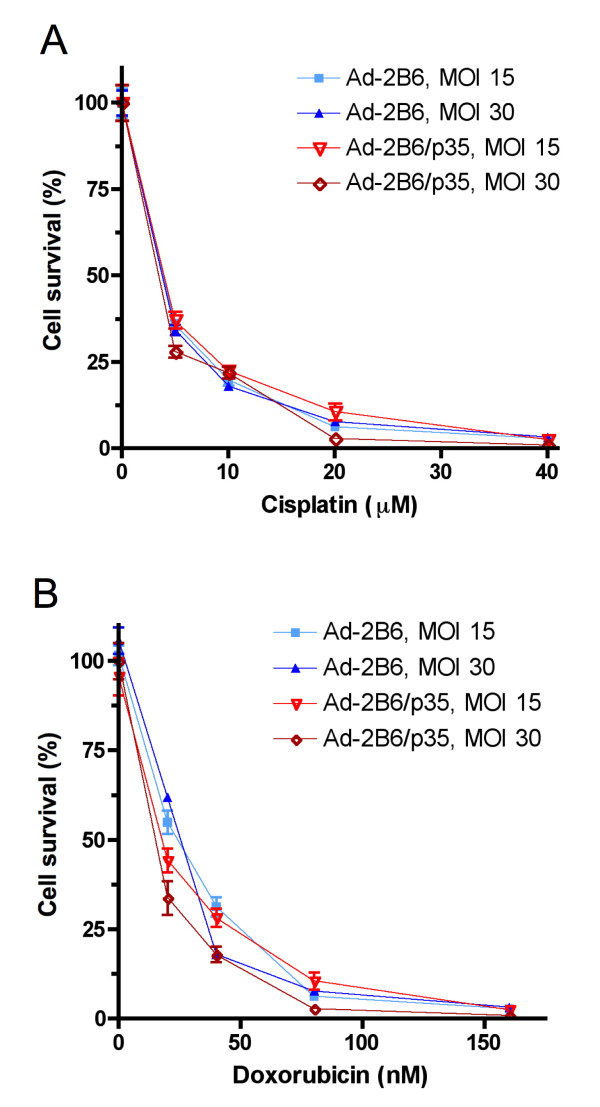
**Chemosensitivity of Adeno-2B6/p35-infected tumor cells to cisplatin and doxorubicin**. Growth inhibition assays showing that Adeno-2B6- and Adeno-2B6/p35-infected U251 cells are equally chemosensitive and can be killed completely by a 4-day continuous exposure to 40 μM cisplatin (A) or 0.16 μM doxorubicin (B).

Since p35 protects cells from CPA-induced cell death, we investigated whether drugs that kill tumor cells by other mechanisms could be used to kill p35-expressing cells that remain viable following CPA treatment. Figure 3 shows that U251 cells infected with either Adeno-2B6 or Adeno-2B6/p35 were equally sensitive to cisplatin (panel A) and doxorubicin (panel B). Thus, U251 tumor cells expressing p35 retain chemosensitivity to other anticancer drugs and do not become globally drug resistant.

### p35 enhances CYP2B6-dependent bystander activity

**Figure 4:**
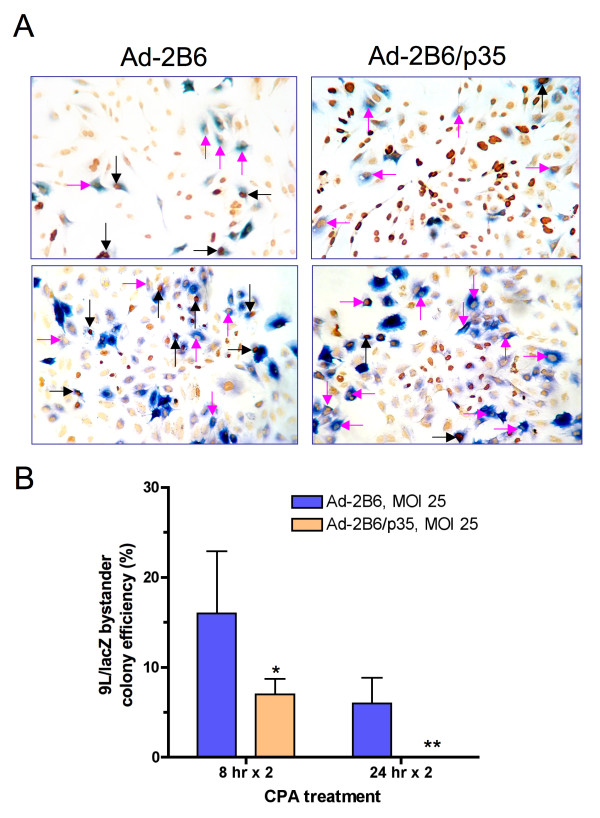
**Adenoviral-delivery of p35 augments CPA bystander activity of Adeno-2B6-infected cultures**. A) Immunostaining of CYP2B6 protein (blue) and TUNEL staining of apoptotic cells (dark brown) in populations of U251 cells infected for 48 hr with Adeno-2B6 (*left photos*) or Adeno-2B6/p35 (*right photos*). Following CPA treatment for 48 hr, apoptotic cells occurred more frequently in the Adeno-2B6/p35-infected culture as compared to the Adeno-2B6-infected culture. In addition, a greater fraction of the CYP2B6-expressing cells (blue) were apoptotic (black arrows) in the Adeno-2B6-infected cultures (34 out of 80 cells (42.5%) versus 11 out of 80 cells (13.8%) for Adeno-2B6/p35). Pink arrows: CYP2B6-expressing cells that are non-apoptotic. B) CPA-induced killing of 9L/lacZ bystander cells, assayed by colony formation activity. A mixed population comprised of 60% U251 cells (readily infectable by adenovirus; serve as P450 prodrug-activating "factory" cells) and 40% 9L/LacZ cells (uninfected at the adenovirus MOI used; serve as bystander cells) was infected for 48 hr by either Adeno-2B6 or Adeno-2B6/p35 at MOI 25. Cells were treated with 1 mM CPA using two different schedules, '8 hr × 2' (two 8 hr CPA treatments, with a 40 hr drug-free period between) or '24 hr × 2' (two 24 hr CPA treatments, with 24 hr drug-free between), modeled based on [30]. The decrease in 9L/lacZ colony formation was 93% and 100% (8 hr × 2 and 24 hr × 2, respectively) in the Adeno-2B6/p35-infected cultures vs. 85% and 94% in the correspondingly treated Adeno-2B6 cultures. Unpaired t-tests were used to determine statistical significance: *: p < 0.05, **: p < 0.001. Data is represented as normalized 9L/lacZ colony formation mean ± SD between three different seeding densities.

Next, we investigated the impact of p35 on the ability of CPA to induce bystander killing by Adeno-2B6-infected tumor cells. First, U251 cells infected with either Adeno-2B6 or Adeno-2B6/p35 were treated with CPA. Cultures were double-stained to identify apoptotic cells (TUNEL staining) and CYP2B6-expressing cells (Figure 4A, brown and blue staining, respectively). Overall bystander killing was increased by p35, as indicated by the higher frequency of apoptotic cells (TUNEL-stained dark brown nuclei) in the cultures infected by Adeno-2B6/p35, as compared to those infected with Adeno-2B6 (Figure 4A, *right *versus *left*). Moreover, a greater fraction of the individual cells expressing CYP2B6 were undergoing apoptosis in the case of the Adeno-2B6-infected cultures (blue + brown double-staining, *black arrows*), as would be expected to occur in the absence of the protective anti-apoptotic effects of p35. In contrast, CYP2B6-expressing cells *not *undergoing apoptosis dominated the Adeno-2B6/p35-infected cultures (*pink arrows*). To further investigate the enhancement of bystander killing by p35, a mixture of 9L/lacZ cells, which are uninfectable at MOIs lower than ~100 (*data not shown*) were seeded in a 40:60 ratio together with U251 cells, which are readily infected by adenovirus. The mixed culture was infected with either Adeno-2B6 or Adeno-2B6/p35 (MOIs based on U251 cell numbers) and then treated with CPA for either two 8 hr or two 24 hr drug treatment cycles [30]. The impact of p35 expression on bystander cytotoxicity was determined in a colony formation assay, where X-gal staining was performed to identify the extent of bystander 9L/lacZ cell survival (Figure 4B). CPA-induced bystander cell killing (decrease in 9L/lacZ colony formation) was substantially more complete in the cultures infected with Adeno-2B6/p35 as compared to Adeno-2B6. Thus, by prolonging the longevity of the CYP2B6-expressing U251 cells, p35 significantly increased CPA-induced bystander cytotoxicity.

### p35-delayed cell death does not impede adenovirus spread

**Figure 5:**
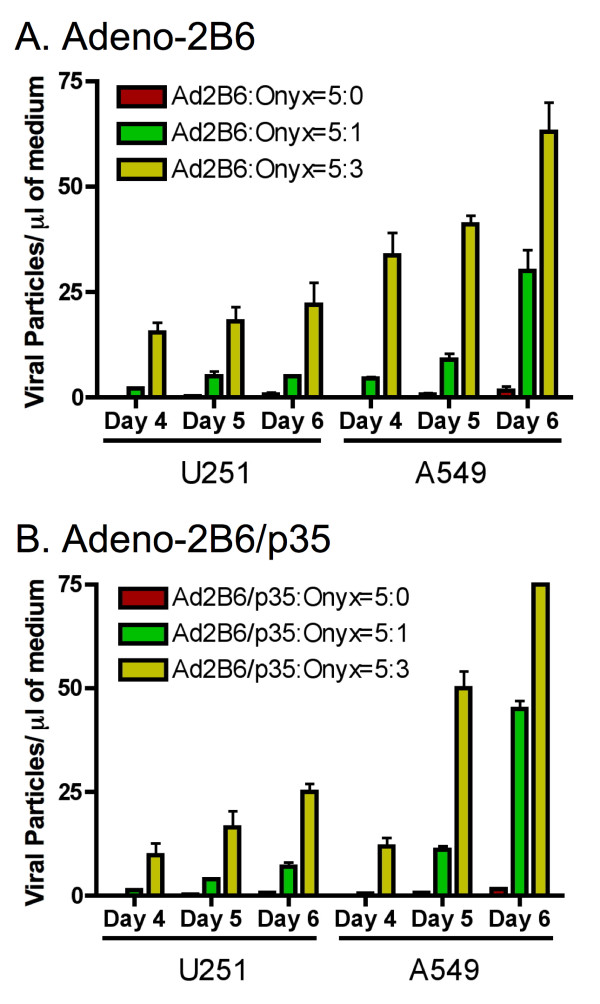
**The apoptosis-inhibiting action of p35 does not slow viral spread**. qPCR assay measuring helper effect of the replication-competent adenovirus ONYX-017 in facilitating spread of either Adeno-2B6 or Adeno-2B6/p35. Viral particle release and spread is similar between Adeno-2B6 (A) and Adeno-2B6/p35 (B) from infected U251 (*left*) and A549 cells (*right*) from day 4 through day 5 post-infection, indicating that the anti-caspase activity of p35 does not interfere with ONYX-017-assisted adenovirus release. The same qPCR primer set for CYP2B6 cDNA was used to determine viral particle release in both panels. qPCR primers for p35 were used to confirm the accuracy of the measured viral particle release in the case of Adeno-2B6/p35 (data not shown). Data shown are mean ± SD, n = 3.

Next, we determined whether the inhibition of apoptosis by p35 interferes with adenoviral spread and expression of a therapeutic transgene. This was accomplished using the cancer cell-replicating adenovirus ONYX-017 as a helper virus [24] to facilitate the spread and expression of the replication-deficient adenoviruses, Adeno-2B6 and Adeno-2B6/p35. U251 and A549 tumor cells were infected with ONYX-017 in combination with either Adeno-2B6 or Adeno-2B6/p35. Cell supernatants were collected and analyzed by qPCR to quantify adenoviral particle release into the culture supernatant. Figure 5 shows that ONYX-017 stimulated a major increase in production and release to the culture supernatant of Adeno-2B6 (panel A) and Adeno-2B6/p35 (panel B), with no significant difference between viruses in the overall pattern and time course of viral release. Thus, the anti-apoptotic activity of p35 does not impede adenoviral release from the infected tumor cells.

### ONYX-017 increases adenovirus p35-associated bystander cytotoxicity

**Figure 6:**
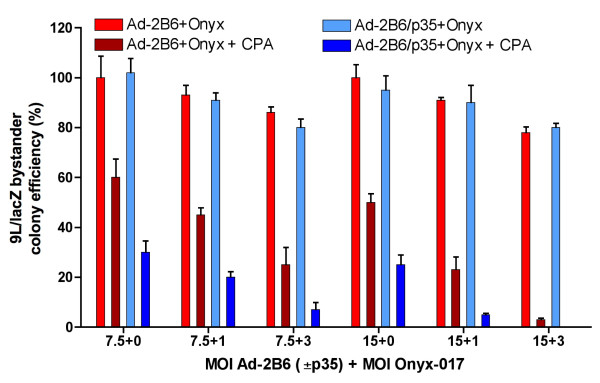
**ONXY-017 helper virus increases p35-enhanced bystander activity**. Colony formation assay of re-seeded 9L/lacZ cells and U251 cells (initially seeded at a 60:40 ratio) following co-infection for 48 hr with ONYX-017 (MOI 0, 1, or 3) and either Adeno-2B6 or Adeno-2B6/p35 (MOI 7.5 or 15) followed by treatment with 1 mM CPA for 48 hr. 9L/lacZ bystander cells were X-gal stained and then quantified by A650 after elution of the stain with DMSO. ONYX-017 conferred helper effects for both Adeno-2B6 and Adeno-2B6/p35, increasing killing of 9L/lacZ bystander cells as a result of the increase in CYP2B6 expression, 4-OH-CPA production and ultimate cell killing. The decrease in 9L/lacZ colony formation, reflecting an increase in bystander activity, was substantially greater with Adeno-2B6/p35 as compared to Adeno-2B6.

Finally, we determined the impact of the helper virus ONYX-017 on the CPA-dependent bystander tumor cell killing by Adeno-2B6/p35. U251 cells seeded together with 9L/lacZ bystander cells in a 40:60 ratio were infected with ONYX-017 in combination with either Adeno-2B6 or Adeno-2B6/p35 at various MOIs. The mixed cultures were then treated with CPA, trypsinized and reseeded. X-gal staining of the resultant individual cell colonies was used to quantify 9L/lacZ cell survival, an indicator of bystander cytotoxicity (Figure 6). At each MOI, the CPA-dependent decrease in 9L/lacZ colony formation was more complete following Adeno-2B6/p35 infection than following Adeno-2B6 infection. Moreover, p35-dependent bystander killing was increased by ONYX-017, as indicated by the decrease in colony formation with increasing ONYX-017 MOIs, from ~30% to ~5% cell survival (Adeno-2B6/p35, MOI 7.5) or from ~25% to 0% cell survival (Adeno-2B6/p35, MOI 15) (Figure 6, dark blue bars). Co-infection with ONYX-017 + Adeno-2B6 also increased CPA-dependent bystander cell killing, most likely by ONYX-017 increasing Adeno-2B6 genomic amplification expression as well as potential spread, resulting in more extensive CPA metabolism and increased 9L/lacZ cell death; however, in all cases bystander cell death was less extensive than that seen with Adeno-2B6/p35. Thus, by facilitating amplification and expression of the replication deficient Adeno-2B6/p35, ONYX-017 further increases the CPA-induced and p35-enhanced bystander activity of CYP2B6.

## Discussion

Gene therapy offers unique opportunities to treat cancers that are either non-responsive or poorly responsive to conventional chemotherapeutic treatments. One approach, termed GDEPT, or suicide gene therapy, holds much promise with low systemic toxicity as a result of tumor-localized prodrug activation following targeted gene delivery with either viral or non-viral vectors [4,31,32]. A key feature of GDEPT is the potential to augment the cytotoxic activity of a prodrug-activating gene by virtue of the bystander killing of nearby or in some cases more distant tumor cells by membrane-diffusible cytotoxic metabolites formed during the course of prodrug activation [23]. Presently, we investigate ways to increase the bystander cytotoxicity of cytochrome P450-based GDEPT [8,22] by inhibiting the caspase 9-dependent apoptotic death [33] that occurs in tumor cells infected with adenovirus expressing the CPA-activating P450 enzyme CYP2B6.

This study addresses a critical limitation of GDEPT, namely, that tumor cells transduced with a prodrug-activating enzyme, when treated with a prodrug, become exposed to high local concentrations of the active drug metabolite and as a consequence die rapidly, thereby halting their ability to continue to activate the prodrug substrate and generate tumor cell toxic drug metabolites. Previously, we reported that stable expression of the pan-caspase inhibitor p35 in 9L gliosarcoma cells engineered to express CYP2B6 delayed, but did not prevent death of the P450-expressing tumor cells, thereby increasing overall CPA activation and cancer cell death [29]. We now report the utility of this strategy when implemented using an adenoviral vector that simultaneously delivers the pan-caspase inhibitor p35 together with CYP2B6 and its electron transfer partner P450 reductase. p35 gene delivery is shown to inhibit CPA-induced apoptosis of the CYP2B6-transduced tumor cells, which in turn leads to increased killing of bystander tumor cells, as verified by the decrease in bystander cell colony survival seen with two different CPA treatment schedules (Figure 4). Importantly, tumor cells expressing p35, although resistant to CPA-induced cell killing, are nevertheless readily killed following exposure to either cisplatin or doxorubicin (Figure 3), whose cell killing mechanism is distinct from that of P450-activated CPA [26]. Moreover, as we have previously shown, tumor cells expressing p35 ultimately die following exposure to activated CPA by a slower, non-apoptotic mechanism [29]. Thus, adenoviral transduction of p35 does not introduce global drug resistance. Moreover, this concept of utilizing an anti-apoptotic factor to delay the death of tumor 'factory cells' to augment therapeutic potency may be extended to other anti-apoptotic factors and other prodrug-activating gene therapy systems as well.

While p35 inhibited CPA-induced apoptosis and delayed tumor cell death, it did not interfere with the helper effect of the cancer replication-conditional adenovirus ONYX-017. This finding may be explained by the fact that adenoviruses typically induce host cell death by an apoptosis-independent mechanism [34] upon expression of the adenoviral death protein [35], which is retained in the E3 region of ONYX-017 [3]. We also show that ONYX-017 is able to enhance bystander cytotoxicity when used in conjunction with Adeno-2B6/p35, and to a lesser extent, Adeno-2B6. This finding is in accordance with earlier studies showing that replication-competent adenoviruses may supply viral replication and repackaging proteins in trans to facilitate the spread and expression of replication-deficient adenoviruses [24]. Overall tumor cell killing was more extensive, as seen in a colony formation assay, when tumor cells were infected with ONYX-017 in combination with Adeno-2B6/p35 as compared to Adeno-2B6. These findings highlight the benefit of using a two-virus helper system for increasing transgene delivery and therapeutic activity. The present approach, involving the expression of CYP2B6, its redox partner P450 reductase, and p35 from a single replication-defective viral vector, may also be extended to cancer cell-replicating viral vectors. Balancing p35-mediated protection and viral lysis and spread from host cells would need to be considered in the context of a one virus system. While replication-competent adenoviruses that directly encode therapeutic transgenes have been investigated [4], a two-virus system such as that described have may be particularly useful in cases where total transgene size is too large to engineer into a single replicating virus.

One potential drawback to the use of adenovirus-delivered p35 in human cancer treatment could be immune responses to the baculovirus-encoded p35, which might eliminate p35-expressing tumor cells and decrease the effectiveness of this strategy. Alternative approaches include the use of other naturally occurring, mammalian anti-apoptotic factors [26] and masking the immunogenicity of adenoviruses and foreign antigens to overcome such issues [36,37]. Further study will be required to investigate these possibilities, in addition to the extension of this approach to other prodrug-activation systems and to gene therapies involving the production of tumor cell toxic protein products, and their translation to the clinic.

## Conclusions

The introduction of p35 into cytochrome P450-based gene therapies constitutes an effective approach to increase bystander killing and overall therapeutic activity. We have shown that the delivery of p35 in combination with P450, and P450 reductase, may be achieved using an adenoviral vector, and that the efficiency of gene delivery does not need to be 100 percent in order to realize significant increases in therapeutic activity. Further studies should investigate the implementation of this strategy using a cancer cell-replicating adenovirus, and its extension to other bystander cytotoxic therapies, including those involving the production of tumor cell toxic protein products.

## Abbreviations

**4-OH-CPA**: 4-hydroxycyclophosphamide; **Adeno**: adenovirus; **CMV**: cytomegalovirus; **CPA**: cyclophosphamide; **CYP2B6**: cytochrome P450 2B6; **FBS**: fetal bovine serum; **GDEPT**: gene-directed enzyme prodrug therapy; **MOI**: multiplicity of infection; **qPCR**: quantitative real-time PCR; **RPMI**: Roswell Park Memorial Institute.

## Competing interests

DJW discloses financial interest in patent applications related to the subject of this study. The authors declare that they have no other competing interests.

## Authors' contributions

JCD organized the data for presentation, carried out statistical analysis and drafted the manuscript, TS and DJW conceived and designed the experiments, TS prepared and characterized the p35-expressing adenovirus and contributed to drafting of the manuscript, and DJW oversaw the overall design and execution of the project and revised the draft manuscript. All authors read and approved the final manuscript.

## Pre-publication history

The pre-publication history for this paper can be accessed here:

http://www.biomedcentral.com/1471-2407/10/487/prepub
